# Acute and Chronic Dopaminergic Depletion Differently Affect Motor Thalamic Function

**DOI:** 10.3390/ijms21082734

**Published:** 2020-04-15

**Authors:** Giuseppe Di Giovanni, Laura Clara Grandi, Ernesto Fedele, Gergely Orban, Agnese Salvadè, Wei Song, Eleonora Cuboni, Alessandro Stefani, Alain Kaelin-Lang, Salvatore Galati

**Affiliations:** 1 Laboratory of Neurophysiology, Department of Physiology and Biochemistry, Faculty of Medicine and Surgery, University of Malta, Msida MSD 2080, Malta; eleonoracuboni@virgilio.it; 2 Neuroscience Division, School of Biosciences, Cardiff University, Cardiff CF10 3AX, UK; 3 Laboratory for Biomedical Neurosciences, Neurocenter of Southern Switzerland, 6900 Taverne, Switzerland; lauraclaragrandi27@gmail.com (L.C.G.); orbangeri@gmail.com (G.O.); agnese.salvade@eoc.ch (A.S.); song.wei@eoc.ch (W.S.); alain.kaelin@eoc.ch (A.K.-L.); 4 Section of Pharmacology and Toxicology, Department of Pharmacy, Center of Excellence for Biomedical Research, University of Genoa, 16148 Genoa, Italy; fedele@difar.unige.it; 5 IRCCS Ospedale Policlinico San Martino, 16132 Genoa, Italy; 6 Department of system medicine, Faculty of Medicine and Surgery, University of Rome “Tor Vergata”, 00133 Rome, Italy; Stefani@uniroma2.it; 7 Medical Faculty, University of Bern, 3008 Bern, Switzerland; 8 Faculty of Biomedical Sciences, Università della Svizzera Italiana, 6900 Lugano, Switzerland; 9 Center for Movement Disorders, Neurocenter of Southern Switzerland, 6900 Lugano, Switzerland

**Keywords:** electrophysiology, microdialysis, immunohistochemistry, L-DOPA, deep brain stimulation

## Abstract

The motor thalamus (MTh) plays a crucial role in the basal ganglia (BG)-cortical loop in motor information codification. Despite this, there is limited evidence of MTh functionality in normal and Parkinsonian conditions. To shed light on the functional properties of the MTh, we examined the effects of acute and chronic dopamine (DA) depletion on the neuronal firing of MTh neurons, cortical/MTh interplay and MTh extracellular concentrations of glutamate (GLU) and gamma-aminobutyric acid (GABA) in two states of DA depletion: acute depletion induced by the tetrodotoxin (TTX) and chronic denervation obtained by 6-hydroxydopamine (6-OHDA), both infused into the medial forebrain bundle (MFB) in anesthetized rats. The acute TTX DA depletion caused a clear-cut reduction in MTh neuronal activity without changes in burst content, whereas the chronic 6-OHDA depletion did not modify the firing rate but increased the burst firing. The phase correlation analysis underscored that the 6-OHDA chronic DA depletion affected the MTh-cortical activity coupling compared to the acute TTX-induced DA depletion state. The TTX acute DA depletion caused a clear-cut increase of the MTh GABA concentration and no change of GLU levels. On the other hand, the 6-OHDA-induced chronic DA depletion led to a significant reduction of local GABA and an increase of GLU levels in the MTh. These data show that MTh is affected by DA depletion and support the hypothesis that a rebalancing of MTh in the chronic condition counterbalances the profound alteration arising after acute DA depletion state.

## 1. Introduction

The motor thalamus (MTh) plays a pivotal role in motor information processing in both physiological and pathological conditions, being directly connected to both the basal ganglia (BG) and the cortex [1]. In particular, the MTh receives the BG input at the level of the ventral-anterior (VA) and the ventral-medial (VM) nuclei [2]. BG are assumed to inhibit the thalamus under basal conditions because substantia nigra pars reticulata (SNr) and internal globus pallidus (GPi) display high spontaneous spiking rates, releasing GABA in the MTh. However, when the BG network is activated by cortical input, BG output is transiently suppressed and downstream targets, including the MTh, are disinhibited [3,4]. Another source of GABA in the MTh comes from the nucleus reticular thalami (NRT) [5], a thin layer of GABAergic cells adjacent to the relay nuclei of the dorsal thalamus, that we have shown to be able to modulate the oscillatory activity of MTh in dopamine (DA)-depleted animals [6]. On the other hand, the excitatory input to the MTh is classically provided by the cerebellothalamic [7] and corticothalamic synapses [8,9]. Moreover, it has been recently shown that some substantia nigra (SN) neurons project to the NRT and the posterior thalamus, and co-release glutamate (GLU) and DA, while the VA and VM would receive preferential GABAergic SN fibers [10]. GABA, GLU and DA, therefore, may interact when modulating synapse and excitability within the MTh in normal and pathological conditions [11,12,13,14]. Although it is well established that the appearance of the cardinal features of Parkinson’s disease (PD) is due to the degeneration of DA neurons, with the contextual dysfunction of different neurotransmitter systems and cortical and BG activity [15,16,17], the evidence of MTh alterations, both at the circuit and cellular level, is scanty and controversial [1,6]. According to the canonical BG schema [18,19], an increase in MTh GABA levels could be the consequence of the DA loss. This agrees with clinical evidence showing that clinical effective sub-thalamic nucleus (STN) deep brain stimulation (DBS) or even levodopa (L-DOPA) in PD patients reversibly reduced the extracellular MTh GABA [20,21]. Nevertheless, a decrease of GABA has been observed in the MTh of 6-OHDA-lesioned rats [22] and certain regions of the thalamus of post-mortem PD brains [23]. Therefore, these discrepancies cannot be only due to the difference in BG circuitry in rats compared to humans.

No changes in GLU concentrations have been described in the thalamuses of PD patients [23], or the striatum or SNr of 6-OHDA-lesioned rats [24]; there was a GLU increase in the entopeduncular nucleus (EPN) [25], in the SNr and the striatum in L-DOPA-induced dyskinesia (LID) 6-OHDA rat [24]. Indeed, AMPA, NMDA and metabotropic GLU receptor antagonists might be a useful target for PD, especially for LID (Cenci et al., 2014).

Finally, the role of the MTh in PD is questioned by the fact that a stereotactic MTh lesion neither worsens Parkinsonian bradykinesia in PD nor regularly causes bradykinesia in patients with essential tremor [26], clearly in contrast to the canonical *box and arrow* schema of BG [18,19].

Thus, the present study was aimed at elucidating the effects of acute and chronic DA depletion, induced respectively by TTX and 6-OHDA [27], on MTh functionality. We, therefore, investigated the electrophysiological changes in the MTh at the level of single neurons from acute and chronic DA-depleted rats. Our main hypothesis was that the MTh is altered differently during rapid changes of DA levels compared to chronic depleted states. This might account for the controversial findings on MTh. Since the BG activity changes in relation to the cortical pattern [28,29], all the recordings were performed during stable cortical slow-wave activity (SWA) induced by urethane anesthesia. This stable cortical pattern allowed us to investigate the coupling of SWA activity with the MTh neuronal activity in acute and chronic DA depletion states. In addition, we measured the extracellular levels of GABA and GLU by microdialysis in the same experimental conditions.

## 2. Results

### 2.1. Effects of DA Depletion on the Activity of the Motor Thalamus Neurons

**Figure 1:**
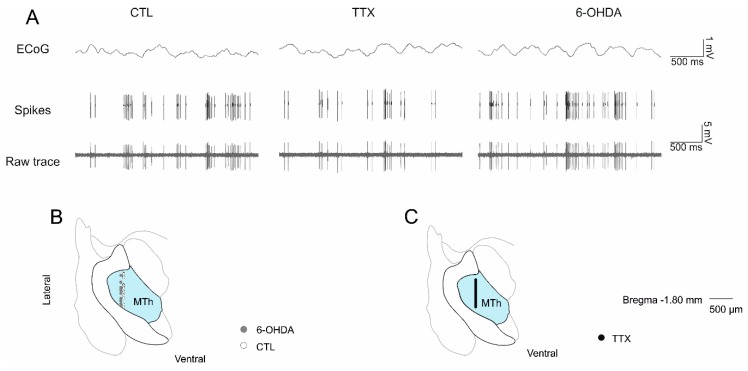
(**A**) Representative electrophysiological recordings of the three groups of animals. From the Table 6. hydroxydopamine (6-OHDA)-lesioned rats, respectively. (**C**) Histological reconstruction of the MTh recording site in tetrodotoxin (TTX)-treated rats (in blue). The black circles represent the recorded neurons in MTh during the TTX-mediated blockade of MFB.

**Figure 2:**
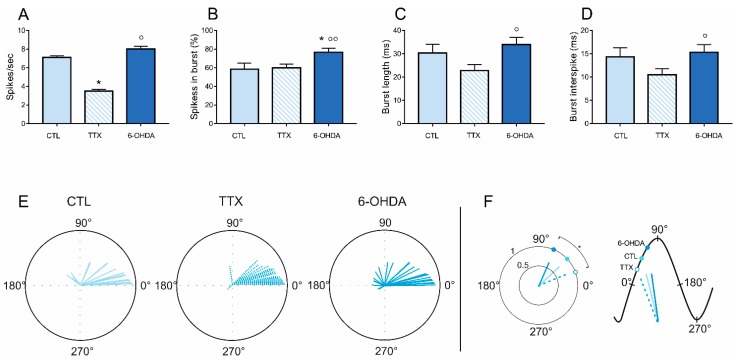
(**A**–**D**) Firing rate and pattern of the MTh in CTL, during tetrodotoxin (TTX) infusion and in 6-hydroxydopamine (6-OHDA)-lesioned rats. * *p* < 0.05. (**E**,**F**). Phase correlation analysis. (**E**) MTh circular plots of the phase-locked firing of the MTh neurons in control, acute and chronic DA depletion states. The vectors of the preferred firing of individual neurons are shown as lines radiating from the center. The last smaller circular plots show the mean vector for the preferred phases of neurons in each group. (**F**) The histograms on the right show the vector lengths and phase angle comparisons between MTh (blue) in control (dark color), acute (dashed color) and chronic (light color) DA depletion states. On the left, the representation of hypothetical cortical slow oscillation comparing the phase angle and vector length between MTh (blue) control (dark color), TTX (dashed color) and 6-OHDA (light color) rats. * *p* < 0.05, Kruskal–Wallis test. MTh: motor thalamus; CTL: control; TTX: tetrodotoxin; 6-OHDA: 6-hydroxydopamine. The 0° represents the mid-ascending phase of cortical slow-wave activity (SWA), the 90° represents the cortical SWA peak and the 180° represents the mid-descending phase of cortical SWA.

We recorded a total of 82 neurons from the MTh (*n* = 23 control, *n* = 25 in 6-OHDA and *n* = 34 in TTX, Figure 1). We found that the mean firing rate of the recorded MTh neurons was lower after TTX infusion (TTX: 3.69 ± 0.18 spike/s, *p* < 0.05) compared to control group (CTL: 7.12 ± 0.15 spikes/s) and 6-OHDA-lesioned rats (6-OHDA: 8.02 ± 0.29 spikes/s; *p* < 0.05) (Figure 2a). The percentage of spikes in burst in chronic 6-OHDA DA-depleted rats (6-OHDA 76.72 ± 4.18%) was higher than in control (CTL 59.89 ± 4.22%, *p* < 0.05) and TTX groups (60.53 ± 6.30%, *p* < 0.01) (Figure 2b). Burst length and interspike of burst (Figure 2c,d) were significantly higher in chronic than in acute TTX-mediated DA depletion but were not different from controls (length of burst: 6-OHDA 34.03 ± 3.01 ms, TTX 22.84 ± 2.61 ms, *p* < 0.05; burst interspike: 6-OHDA 15.33 ± 1.65 ms, TTX 10.5 ± 1.29 ms).

### 2.2. Effects of DA Depletion on the Cortical–Thalamic Coupling

We compared the preferred phase angles and the vector lengths in control, acute and chronic DA depletion states. In control rats, the MTh neurons fired preferentially at 42.77 ± 12.36° (*n* = 21, Figure 2e), with a vector length of 0.73 ± 0.06. In acute DA-depleted animals (*n* = 34), the phase was 22.54 ± 6.96° with a vector length of 0.83 ± 0.03, while in the chronic DA depletion state (*n* = 22), it was 79.89 ± 15.98° with a vector length of 0.56 ± 0.07. The vector length, i.e., the strength of corticothalamic coupling, was significantly lower in chronic DA depletion state than in control and in acute DA depletion state (*p* < 0.05). The phase angle was significantly lower in control and acute DA depletion state than that in chronic DA depletion state (*p* < 0.05) (Figure 2e).

### 2.3. Effects of DA Depletion on the Extracellular Levels of GABA and GLU in the Motor Thalamus

**Figure 3:**
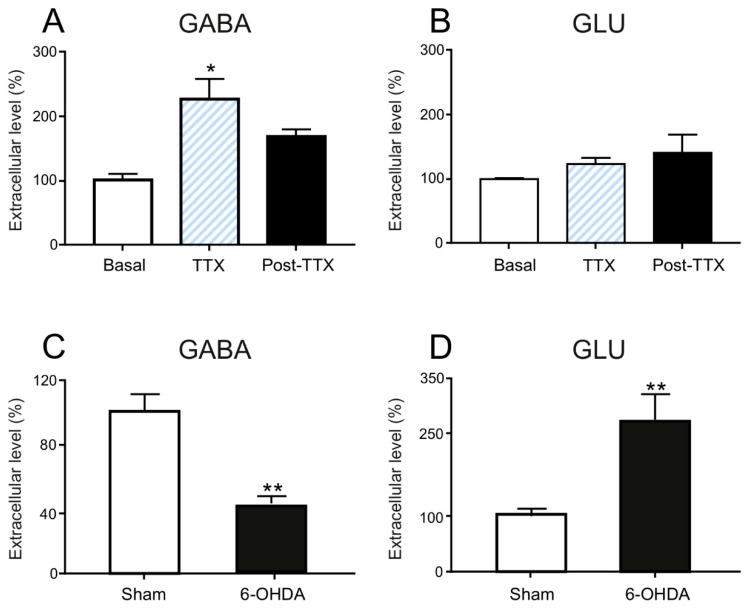
Effect of acute (TTX) and chronic (6-OHDA) dopamine (DA)-depletion on the extracellular levels of endogenous GABA and glutamate (GLU) in the motor thalamus (MTh). TTX infused in the medial forebrain bundle induced an increase in the endogenous GABA extracellular levels (**A**) while did not change the GLU levels in the MTh. Data in (**A**) and (**B**) are expressed as mean ± SEM normalized to the baseline levels (pre TTX infusion) from *n* = 14 experiments per group; * *p* < 0.05. One-and two-way ANOVA for repeated measures, followed by Dunnet’s multiple comparison test. 6-OHDA-lesion infused in the medial forebrain bundle produced, after 2 weeks, a decrease in GABA (**C**) and an increase in GLU extracellular levels in the MTh (**D**). Data in (**C**) and (**D**) are expressed as means ± SEMs normalized to sham-lesioned (*n* = 9) neurotransmitter levels from *n* = 9 experiments per group; ** *p* < 0.01, unpaired student *t*-test.

ANOVA analysis followed by Dunnet’s multiple comparison test showed a significant effect of TTX infused in the medial forebrain bundle induced an increase in the endogenous GABA extracellular level (215 ± 67.5; *n* = 14; *p* < 0.05) compared to the control (100 ± 7.90, *n* = 14) and post-infusion period (159.2 ± 50.2, *n* = 14) (Figure 3a). No changes in the GLU levels were observed in the MTh after the infusion of TTX (PRE: 100 ± 2.69; TTX: 122.7 ± 24.8, *n* = 7; POST: 123.7 ± 53.9, *n* = 14) (Figure 3b). The extracellular GABA levels in the MTh were significantly lower in 6-OHDA animals than in sham-lesioned animals (100 ± 11.09 vs. 43.54 ± 6.65, *n* = 9; ** *p* < 0.01) (Figure 3c). GLU levels were significantly augmented in the MTh of 6-OHDA rats when compared to those measured in sham-lesioned rats by unpaired student t-test (100 ± 13.75 vs. 276.69 ± 43.52, *n* = 9; ** *p* < 0.01) (Figure 3d) levels.

## 3. Discussion

We demonstrated that MTh is deeply affected by both acute and chronic DA depletion both in terms of electrophysiological and neurochemical alterations. The main findings of the present study are threefold. Firstly, the results emphasize the different effects of DA deafferentation on MTh, with a decrease in firing rate in TTX-treated animals and an increase of burst firing in 6-OHDA-lesioned rats. Secondly, the results show that DA depletions change, in an opposite fashion, MTh-cortical coupling with the MTh neurons fired preferentially at the beginning of the UP-state in TTX-treated rats compared to the 6-OHDA-lesioned rats, which showed a shift towards the peak of the SWA. Finally, the neurochemical results demonstrated that the extracellular levels of GABA increase and those of GLU do not change in the MTh of TTX-treated animals; in the 6-OHDA-lesioned rats, GABA levels decreased and those of GLU increased.

Despite representing a crucial point in the cortico-BG circuit, only a few studies have investigated the role of the MTh and its properties in PD, and controversial data are present in the literature [1,6]. Furthermore, controversial data are present in the literature which regard MTh electrophysiology and properties in the Parkinsonian state. While some studies show a slight MTh firing rate reduction in awake Parkinsonian monkeys [30,31,32,33,34], others revealed increased [1] or unchanged firing rates [35]. We recently showed that all the EEG frequency bands are altered in TTX and 6-OHDA-depleted states; MTh oscillation changes occur preferentially in acute DA depletion, but not in the chronic state [6].

In the present study, we demonstrated that the TTX-inhibition of the medial forebrain bundle (MFB) (for technical details, see our review [27]) determined an acute increase in GABA content with the reduction of the MTh activity. In rats, the SNr activity is reduced after acute DA depletion caused by TTX [36], in accordance with electrophysiological and biochemical data obtained in PD patients [37]. Thus, the changes in MTh activity have to be due to other GABAergic afferents of the network. For instance, we occasionally recorded NRT neurons during TTX infusion and observed a consistent increment of their activity (unpublished observation). Considering that NRT is a powerful modulator of different high-order thalamic nuclei [38], it is likely that this nucleus tightly controls the MTh as well. Conversely, we observed that the MTh neuronal firing rate returns to control condition in the chronic depletion state induced by 6-OHDA. This could be due to the aberrant decrease in GABA and an increase in GLU levels in the MTh of 6-OHDA-lesioned rats that might compensate for the marked inhibition observed in the acute depletion state. The shift of the excitation/inhibition (E/I) balance toward a net excitation in the MTh of the 6-OHDA lesioned rats might indicate that the plastic changes observed in the striatum and cortex of the PD animal model [39] could also occur at the level of the thalamic area. Surprisingly, GABA levels decrease in the chronic DA-depleted MTh, a finding that cannot be explained by the canonical BG/thalamus/cortex loop [19]. Nevertheless, our data, although surprising, are in line with the findings of the only available in vivo study showing a decrease of GABA in the MTh of rats lesioned by striatal infusion of 6-OHDA [22]. Instead, we are the first to report a sustained GLU increase in the MTh of animal models of PD. The decrease in GABA levels is not trivial since the administration of the vasoactive intestinal peptide (VIP) was effective at reversing the motor deficits and contextually increased thalamic GABA contents [22]. Other areas may be involved in rebalancing the MTh activity in chronic DA depletion, such as hyperactivity of the cortex, likely leading the increased release of GLU that we observed within the MTh of 6-OHDA-lesioned rats. The increase of burst activity in the MTh that we recorded in 6-OHDA conditions might depend on the cortex, which, by acting as the main driver, produces a burst input, leading to a coincident burst of activity in MTh neurons, without necessarily causing LTS bursts [1]. The fact that we did not observe a strong effect on burst activity may depend on the canceling effects of different inputs to the MTh, such as synchronized bursts from the BG. The contextual increase in GLU may also cause the production of nitric oxide and contribute to PD development processes [15,40,41].

However, it is also necessary to underline that, since the lesion in our study was unilateral, we cannot exclude that the contralateral hemisphere could compensate, at least partially, for these changes [42]. Furthermore, a recent tractography study in healthy subjects showed that MTh may receive DAergic projections from the ventral tegmental area that are directed towards the supplementary motor area and primary motor cortex (44).

An interesting finding of this study is the interplay between motor thalamus and cortical activity. The DA depletion influenced mainly the MTh-cortical coupling. The divergence of MTh-SWA coupling in the chronic state of DA depletion further suggests that the MTh has undergone the effect of compensatory mechanisms, changing the cross-talk between MTh and the cortex. In accordance with the SWA pattern, the MTh neurons fired preferentially at the beginning of the UP-state in TTX-treated rats compared to the 6-OHDA-lesioned rats, which showed a shift towards the peak of the SWA. Thus, in 6-OHDA rats, there is a delay in the coupling between neuronal activity and the beginning of cortical SWA

We showed that, following the acute reduction of DA, the MTh immediately changes the activity, but the chronic lack of DA leads other structures to rebalance the MTh activity. It is possible to hypothesize that, inside the complex circuit involved in PD, rapid clinical transitions of the motor and non-motor symptoms could be directly related to rapid changes in MTh activity, as observed during DBS surgery [20,21]. These hypotheses need further investigation of the MTh activity in PD animal models and PD patients.

Our study presents a number of limitations. For instance, we should keep in mind that our recordings and the position of the microdialysis probe were at ranges of depths in the MTh that correspond to both basal ganglia and cerebellar recipient zones. Therefore, our electrophysiological and neurochemical data may have included values obtained from the two different MTh territories. Moreover, we did not distinguish the two types of MTh neurons during cortical activation by performing the tail-pinch (Nakamura et al., Cerebral cortex, 2014). Further experiments will be required to identify the tonic and burst firing cells in MTh in response to cortical activation, and the eventual changes of their firing characteristics following DA depletion by TTX and 6-OHDA treatment. Another limitation of this study is the use of toxin-induced PD animal models and anesthetized preparations. Despite the usefulness of the animal models, they have limitations that need to be considered when interpreting our findings and translating them to humans [27]. Nevertheless, although rodent models cannot recapitulate all features of PD, they remain a crucial approximation in order to open new avenues.

In conclusion, our data show that in chronic conditions, the firing changes observed in the MTh are relatively mild, considering the profound changes observed in other BG nuclei. This could be due to remodeling and adaptation developed as a consequence of the DA depletion. Indeed, acute TTX induced a strong decrease in the firing rate of MTh neurons. On the other hand, the modification of GLU and GABA are highly significant in 6-OHDA DA-depleted rats and are likely a consequence of changes in the MTh inputs, difficult to explain with the current knowledge of PD pathology. The roles of the MTh in the mechanisms of information processing under physiological and pathological conditions need to be further investigated and clarified.

## 4. Materials and Methods

### 4.1. Animals

Most of the electrophysiological, neurochemical and histological procedures were carried out on adult male Sprague-Dawley rats in compliance with Swiss laws on animal experimentation, and all procedures were approved by the Animal Research Committee and the Veterinary Office of the Canton of Ticino, Switzerland (TI-08-2015).

A subset of neurochemical experiments was performed on male Sprague-Dawley rats at the Department of Physiology and Biochemistry at the University of Malta in accordance with the European Union (EU) Directive 2010/63/EU and the Institutional Animal Use and Care Committee (IAUCC) of the University of Malta.

Utmost care was taken to limit the number of rats used and their suffering.

### 4.2. Pharmacological Blockade of the Medial Forebrain Bundle

The pharmacological blockade of the MFB was performed on adult male Sprague–Dawley rats weighing 399 ± 68.4 g according to our previously described method [6,27]. Briefly, TTX was infused via reverse microdialysis using a probe with a 1 mm dialytic membrane (CMA/11 microdialysis probe, CMA Microdialysis, Stockholm, Sweden), which was positioned according to the following coordinates: 2.5 mm posterior to the bregma, 2 mm lateral to the midline and 8.6 mm below the cortical surface [43]. The probe was perfused with saline solution for 1 h and then with TTX (1 µM) using a syringe pump (CMA/400, CMA Microdialysis) at a flow rate of 1 µL/min. The MTh neuronal activity was recorded and analyzed for at least the next 10 min after the TTX infusion in 27 animals, while the GABA and GLU MTh levels were measured in 14 rats.

### 4.3. Unilateral 6-OHDA Lesion

We performed standard unilateral 6-OHDA denervation in the left hemisphere [6,27] to obtain chronic DA-depleted animals. The animals (399 ± 36.3 g) were anesthetized with 1.5%–2.5% isoflurane in oxygen and then injected with desipramine (25 mg/kg, i.p.) and pargyline (50 mg/kg, i.p.) to minimize 6-OHDA uptake by noradrenergic neurons and metabolism by MAO, respectively. Then, the animals were mounted on a stereotaxic instrument (Stoelting Co., Wheat Lane, Wood Dale, IL, USA) for injection of the neurotoxin (6-OHDA; Sigma; 8 µg/4 µL of saline solution containing 0.1% of ascorbic acid) in the medial forebrain bundle (MFB; stereotaxic coordinates: 2.5 mm posterior to the bregma, 2 mm lateral to the midline, and 8.6 mm below the cortical surface) [44]. The MTh neuronal activity was recorded an analyzed in 6-OHDA-lesioned rats (*n* = 8) and control animals (*n* = 6). The microdialysis experiments were performed in sham animals (*n* = 9), receiving instead, 4 µL of saline solution containing 0.1% of ascorbic acid infused into the MFB and 6-OHDA-lesioned animals (*n* = 9). Electrophysiological and microdialysis experiments were performed 21–29 days after the administration of 6-OHDA

### 4.4. Prerecording Surgery

Rats were anesthetized with urethane (1.4 g/kg, i.p.) (Sigma Chemical Co., St Louis, MO, USA) and mounted on a stereotaxic instrument (Stoelting Co.) according to our standard operating procedure [6,27]. Body temperature was maintained at 37–38 °C with a heating pad (Stoelting Co.). After a local anaesthetic injection (lidocaine 0.5%), a midline scalp incision was made, and the skull was almost completely drilled on the left side. The dura were then spread out to expose the cortical surface.

### 4.5. Electrophysiological Recordings

We recorded single neurons for the entire dorsoventral track in the same medio-lateral site in the MTh. In particular, we recorded a total of 82 neurons from the MTh (*n* = 23 control, *n* = 25 in 6-OHDA and *n* = 34 in TTX) from 5000 µm from the cortex to a maximum of 5770 µm.

We recorded multiple MTh neurons from TTX infused rats over the 10 min after infusion The MTh neurons (*n* = 34) recorded after the TTX-mediated blockade of the MFB were localized from 5250 µm to 5870 µm from the first neuronal detection of the cortex.

The MTh neurons recorded were localized at the following coordinates: 1.8 mm posterior and 2.2 medial to bregma. Single unit activity was off-line analyzed using Spike 2 software (Cambridge Electronic Design, Cambridge, UK) and principal component analysis for spike sorting.

The Electrocorticogram (ECoG) was recorded through a screw electrode (Dentorama, 8 mm of total length, 3 mm tip length) placed on the cortical surface above the frontal cortex (3.0 mm anterior of bregma and 2.0 mm lateral to the midline) and referenced against an indifferent screw electrode placed above the cerebellum. Raw ECoG was bandpass filtered (0.1–300 Hz) and amplified (×2000; Neurolog). The ECoG was on-line digitalized at a sample rate of 600 kHz through an analogical/digital interface (Micro1401 mk II, Cambridge Electronic Design, Cambridge, UK) and stored on a computer to identify off-line robust SWA, which will be the object of further analysis. Simultaneously, we collected the single-unit activity from the MTh. The acquisition of the recording was performed after the stabilization of neuronal activity. The recordings were performed using a tungsten electrode (WPI, TM33B01; impedance 0.9–1.2 MΩ). The trace containing the neuron action potentials was amplified (×10,000; Neurolog), bandpass filtered (300–1000 Hz) and sampled at 60 kHz for digital storage on a computer connected to the CED 1401 interface [36]. The neuronal activity was recorded for at least 10 min after the TTX infusion.

Figure 1A shows an example of the recordings with the EEG traces, i.e., SWA (top panel); the discriminated single neurons (middle panel); and the neuronal traces (bottom panel).

### 4.6. Microdialysis Analysis

An additional series of experiments were conducted on adult rats (312 ± 25 g) to measure GABA and GLU before, during and after the TTX-mediated blockade of MFB and in sham and 6-OHDA-lesioned rats [28]. Microdialysis was performed using concentric microdialysis probes (CMA11 with 1 mm PES membrane, outside diameter 0.6 mm, cut-off 35 kDa, Microbiotech) which were positioned into the right MTh (1.8 mm posterior to the bregma, 2 mm lateral to the midline and 6.2 mm below the cortical surface).

Probes were then connected to a high precision micro-injection pump (CMA/100, CMA 159 Microdialysis) and infused at a flow rate of 5 μL/min with an artificial cerebrospinal fluid containing 160 (in mM): NaCl 145, KCl 3, MgCl2 1, CaCl2 1.26, buffered at pH 7.4 with 2 mM phosphate buffer.

After stabilization (4 h), consecutive samples were collected every 10 min. In the acute depleted animals, TTX was infused in the MFB (see above) at the end of the 3rd sample. All samples were immediately frozen at −80 °C until analyzed. At the end of the experiment, rats were sacrificed, and the correct position of the probe was histologically verified (36).

For the experiments in 6-OHDA and sham-lesioned animals, the microdialysis experiments were performed 21–29 days after the lesion.

The concentrations of GABA and GLU in the samples were determined by HPLC analysis following precolumn derivatization with o-phthalaldehyde and resolution through a C18-reverse phase chromatographic column (Chrompack, Middleburg, The Netherlands) connected to a fluorometric detector (excitation 350 nm; emission 450 nm). Buffers were prepared as follows: solvent A, 0.1 M sodium acetate (pH 5.8): methanol, 80:20; solvent B, 0.1 M sodium acetate (pH 5.8): methanol, 20:80; solvent C, 0.1 M sodium acetate (pH 6.0): methanol, 80:20. Gradient programme: 100% C for 4 min from the initiation of the programme; 90% A and 10% B in 1 min; isocratic step for 2 min; 78% A and 22% B in 2 min; isocratic step 172 for 6.50 min; 66% A and 34% B in 1.10 min; isocratic step for 1.50 min; 42% A and 58% B in 1.10 min; isocratic step for 3.50 min; flow rate 0.9 mL/min (Waters 600MS gradient system). Homoserine was used as an internal standard [21].

### 4.7. Histological and Microscopic Analysis

At the end of the experiment, the positions of the electrodes (Figure 1b,c) were histologically checked, and the unilateral dopaminergic lesion in each PD rat was confirmed by quantification of tyrosine hydroxylase (TH) immunoreactivity, as described and analyzed in our previous publication [28].

### 4.8. Statistical Analysis

Concerning the single-unit data, statistical comparisons of firing rates and burst parameters (control vs. 6-OHDA and post-TTX) were conducted using the Kruskal–Wallis test with the Man–Whitney test for post hoc comparisons (asymptotic significance (2-tailed) *p*-value).

The firing rate comparison was performed taking into account the mean of the first 5 min of recording. The comparison of the burst activity was performed in selected 100-s increments in the presence of stable cortical urethane-induced SWA with a frequency below 3 Hz, as shown elsewhere (45.) SWA is not affected by DA denervation and DAergic treatment [45].

To investigate the correlation between cortex and MTh, we performed the phase correlation analysis, with the calculation of descriptive circular statistics being performed using the CircStat toolbox for MATLAB (2). For the burst analysis, the phase correlation analysis was performed in the same selected 100 s in the presence of stable cortical SWA. For circular analyses and statistical comparisons, we included all neurons from the previous analyses.

For each neuron, the linear phase histograms were analyzed, taking into account the up phase of the SWA, and the spike phase values were used for circular analysis. Then, each neuron was tested for significant SWA phase-locked firing (comparison of degree; Rayleigh’s uniformity test). The selected neurons were then used for the analysis of the preferred phase angle, i.e., the mean resultant vector in degree (with 90° corresponding to the peak of the SWA phase), and the vector length (corresponding to the level of dispersion of the firing). The mean resultant vector length of the phase distribution, bound between zero and one, was used to quantify the level of phase-locking around the mean angle (the closer to one, the more grouped the firing; 2).

The differences between the mean of the resultant vector (degree) and the vector lengths of the three groups (Control, TTX and 6-OHDA) of MTh were tested using the Kruskal-Wallis test.

All circular analyses were performed using the degree and the upper peak in SWA oscillations corresponding to a 90° phase and the down peak to a 270° phase.

In all experimental conditions, all neurons were tested for significantly phase-locked firing; i.e., not randomly distributed in relation to the SWA (comparison of degree; Rayleigh’s uniformity test) according to the method described by Nakamura and colleagues [2]. Firstly, we analyzed the activity by using the CircStat toolbox for MATLAB. As reported in the text, for each neuron, the linear phase histograms were analyzed, taking into account the up phase of the SWA, and the spike phase values were used for circular analysis. Then, each neuron was tested for significant SWA phase-locked firing; i.e., not-randomly distributed in relation to the SWA (comparison of degree; Rayleigh’s uniformity test). To exclude this hypothesis, we performed chi-square tests on the percentage of discarded MTh neurons in the three conditions; i.e., control, acute and chronic DA depletion states. The significance threshold was set at *p* < 0.05 for all analyses.

## Abbreviations

MTh	motor thalamus
DA	dopamine
GLU	glutamate
*GABA*	*gamma*-aminobutyric acid
TTX	tetrodotoxin
6-OHDA	6-hydroxydopamine
VA	ventral-anterior thalamus
VM	ventral-medial
SNr	substantia nigra pars reticulata
GPi	internal globus pallidus
NRT	nucleus reticular thalami
SN	substantia nigra
substantia	substantia nigra pars compacta
PD	Parkinson’s disease
STN	sub-thalamic nucleus
DBS	deep brain stimulation
L-DOPA	levodopa
*HT2A*	entopeduncular nucleus
EPN	immunohistochemistry
LID	L-DOPA-induced dyskinesia
SWA	slow-wave activity
MFB	medial forebrain bundle

## References

[1] Bosch-Bouju C., Hyland B.I., Parr-Brownlie L.C. (2013). Motor thalamus integration of cortical, cerebellar and basal ganglia information: Implications for normal and parkinsonian conditions. Front. Comput. Neurosci..

[2] Nakamura K.C., Sharott A., Magill P.J. (2014). Temporal coupling with cortex distinguishes spontaneous neuronal activities in identified basal ganglia-recipient and cerebellar-recipient zones of the motor thalamus. Cereb. Cortex.

[3] Wichmann T., Kliem M.A. (2004). Neuronal activity in the primate substantia nigra pars reticulata during the performance of simple and memory-guided elbow movements. J. Neurophysiol..

[4] Avila I., Parr-Brownlie L.C., Brazhnik E., Castaneda E., Bergstrom D.A., Walters J.R. (2010). Beta frequency synchronization in basal ganglia output during rest and walk in a hemiparkinsonian rat. Exp. Neurol..

[5] Pare D., Steriade M., Deschenes M., Oakson G. (1987). Physiological characteristics of anterior thalamic nuclei, a group devoid of inputs from reticular thalamic nucleus. J. Neurophysiol..

[6] Grandi L.C., Kaelin-Lang A., Orban G., Song W., Salvade A., Stefani A., Di Giovanni G., Galati S. (2018). Oscillatory Activity in the Cortex, Motor Thalamus and Nucleus Reticularis Thalami in Acute TTX and Chronic 6-OHDA Dopamine-Depleted Animals. Front. Neurol..

[7] Yamamoto T., Noda T., Miyata M., Nishimura Y. (1984). Electrophysiological and morphological studies on thalamic neurons receiving entopedunculo- and cerebello-thalamic projections in the cat. Brain Res..

[8] Kakei S., Na J., Shinoda Y. (2001). Thalamic terminal morphology and distribution of single corticothalamic axons originating from layers 5 and 6 of the cat motor cortex. J. Comp. Neurol..

[9] Kultas-Ilinsky K., Sivan-Loukianova E., Ilinsky I.A. (2003). Reevaluation of the primary motor cortex connections with the thalamus in primates. J. Comp. Neurol..

[10] Antal M., Beneduce B.M., Regehr W.G. (2014). The substantia nigra conveys target-dependent excitatory and inhibitory outputs from the basal ganglia to the thalamus. J. Neurosci..

[11] Yague J.G., Cavaccini A., Errington A.C., Crunelli V., Di Giovanni G. (2013). Dopaminergic modulation of tonic but not phasic GABAA-receptor-mediated current in the ventrobasal thalamus of Wistar and GAERS rats. Exp. Neurol..

[12] Lavin A., Grace A.A. (1998). Dopamine modulates the responsivity of mediodorsal thalamic cells recorded in vitro. J. Neurosci..

[13] Carlsson M., Carlsson A. (1990). Interactions between glutamatergic and monoaminergic systems within the basal ganglia--implications for schizophrenia and Parkinson’s disease. Trends Neurosci..

[14] Munoz M.D., de la Fuente N., Sanchez-Capelo A. (2020). TGF-beta/Smad3 Signalling Modulates GABA Neurotransmission: Implications in Parkinson’s Disease. Int. J. Mol. Sci..

[15] Esposito E., Di Matteo V., Di Giovanni G. (2007). Death in the substantia nigra: A motor tragedy. Expert Rev. Neurother..

[16] Hammond C., Bergman H., Brown P. (2007). Pathological synchronization in Parkinson’s disease: Networks, models and treatments. Trends Neurosci..

[17] Boraud T., Bezard E., Bioulac B., Gross C.E. (2002). From single extracellular unit recording in experimental and human Parkinsonism to the development of a functional concept of the role played by the basal ganglia in motor control. Prog. Neurobiol..

[18] Albin R.L., Young A.B., Penney J.B. (1989). The functional anatomy of basal ganglia disorders. Trends Neurosci..

[19] DeLong M.R. (1990). Primate models of movement disorders of basal ganglia origin. Trends Neurosci..

[20] Stefani A., Fedele E., Vitek J., Pierantozzi M., Galati S., Marzetti F., Peppe A., Bassi M.S., Bernardi G., Stanzione P. (2011). The clinical efficacy of L-DOPA and STN-DBS share a common marker: Reduced GABA content in the motor thalamus. Cell Death Dis..

[21] Stefani A., Fedele E., Pierantozzi M., Galati S., Marzetti F., Peppe A., Pastore F.S., Bernardi G., Stanzione P. (2011). Reduced GABA Content in the Motor Thalamus during Effective Deep Brain Stimulation of the Subthalamic Nucleus. Front. Syst. Neurosci..

[22] Korkmaz O.T., Tuncel N., Tuncel M., Oncu E.M., Sahinturk V., Celik M. (2010). Vasoactive intestinal peptide (VIP) treatment of Parkinsonian rats increases thalamic gamma-aminobutyric acid (GABA) levels and alters the release of nerve growth factor (NGF) by mast cells. J. Mol. Neurosci..

[23] Gerlach M., Gsell W., Kornhuber J., Jellinger K., Krieger V., Pantucek F., Vock R., Riederer P. (1996). A post mortem study on neurochemical markers of dopaminergic, GABA-ergic and glutamatergic neurons in basal ganglia-thalamocortical circuits in Parkinson syndrome. Brain Res..

[24] Robelet S., Melon C., Guillet B., Salin P., Kerkerian-Le Goff L. (2004). Chronic L-DOPA treatment increases extracellular glutamate levels and GLT1 expression in the basal ganglia in a rat model of Parkinson’s disease. Eur. J. Neurosci..

[25] Biggs C.S., Starr M.S. (1997). Dopamine and glutamate control each other’s release in the basal ganglia: A microdialysis study of the entopeduncular nucleus and substantia nigra. Neurosci. Biobehav. Rev..

[26] Marsden C.D., Obeso J.A. (1994). The functions of the basal ganglia and the paradox of stereotaxic surgery in Parkinson’s disease. Brain A J. Neurol..

[27] Grandi L.C., Di Giovanni G., Galati S. (2018). Animal models of early-stage Parkinson’s disease and acute dopamine deficiency to study compensatory neurodegenerative mechanisms. J. Neurosci. Methods.

[28] Galati S., Stanzione P., D’Angelo V., Fedele E., Marzetti F., Sancesario G., Procopio T., Stefani A. (2009). The pharmacological blockade of medial forebrain bundle induces an acute pathological synchronization of the cortico-subthalamic nucleus-globus pallidus pathway. J. Physiol..

[29] Prosperetti C., Di Giovanni G., Stefani A., Moller J.C., Galati S. (2013). Acute nigro-striatal blockade alters cortico-striatal encoding: An in vivo electrophysiological study. Exp. Neurol..

[30] Voloshin M., Lukhanina E.P., Kolomietz B.P., Prokopenko V.F., Rodionov V.A. (1994). Electrophysiological investigation of thalamic neuronal mechanisms of motor disorders in parkinsonism: An influence of D2ergic transmission blockade on excitation and inhibition of relay neurons in motor thalamic nuclei of cat. Neuroscience.

[31] Schneider J.S., Rothblat D.S. (1996). Alterations in intralaminar and motor thalamic physiology following nigrostriatal dopamine depletion. Brain Res..

[32] Vitek J.L. (2002). Mechanisms of deep brain stimulation: Excitation or inhibition. Mov. Disord. Off. J. Mov. Disord. Soc..

[33] Ni Z.G., Gao D.M., Benabid A.L., Benazzouz A. (2000). Unilateral lesion of the nigrostriatal pathway induces a transient decrease of firing rate with no change in the firing pattern of neurons of the parafascicular nucleus in the rat. Neuroscience.

[34] Kammermeier S., Pittard D., Hamada I., Wichmann T. (2016). Effects of high-frequency stimulation of the internal pallidal segment on neuronal activity in the thalamus in parkinsonian monkeys. J. Neurophysiol..

[35] Pessiglione M., Guehl D., Rolland A.S., Francois C., Hirsch E.C., Feger J., Tremblay L. (2005). Thalamic neuronal activity in dopamine-depleted primates: Evidence for a loss of functional segregation within basal ganglia circuits. J. Neurosci..

[36] Galati S., D’Angelo V., Olivola E., Marzetti F., Di Giovanni G., Stanzione P., Stefani A. (2010). Acute inactivation of the medial forebrain bundle imposes oscillations in the SNr: A challenge for the 6-OHDA model?. Exp. Neurol..

[37] Galati S., Mazzone P., Fedele E., Pisani A., Peppe A., Pierantozzi M., Brusa L., Tropepi D., Moschella V., Raiteri M. (2006). Biochemical and electrophysiological changes of substantia nigra pars reticulata driven by subthalamic stimulation in patients with Parkinson’s disease. Eur. J. Neurosci..

[38] Crick F. (1984). Function of the thalamic reticular complex: The searchlight hypothesis. Proc. Natl. Acad. Sci. USA..

[39] Calabresi P., Mercuri N.B., Di Filippo M. (2009). Synaptic plasticity, dopamine and Parkinson’s disease: One step ahead. Brain A J. Neurol..

[40] Di Matteo V., Benigno A., Pierucci M., Giuliano D.A., Crescimanno G., Esposito E., Di Giovanni G. (2006). 7-nitroindazole protects striatal dopaminergic neurons against MPP+-induced degeneration: An in vivo microdialysis study. Ann. N. Y. Acad. Sci..

[41] Di Matteo V., Pierucci M., Benigno A., Esposito E., Crescimanno G., Di Giovanni G. (2010). Critical role of nitric oxide on nicotine-induced hyperactivation of dopaminergic nigrostriatal system: Electrophysiological and neurochemical evidence in rats. CNS Neurosci. Ther..

[42] Pierucci M., Di Matteo V., Benigno A., Crescimanno G., Esposito E., Di Giovanni G. (2009). The unilateral nigral lesion induces dramatic bilateral modification on rat brain monoamine neurochemistry. Ann. N. Y. Acad. Sci..

[43] Hosp J.A., Coenen V.A., Rijntjes M., Egger K., Urbach H., Weiller C., Reisert M. (2019). Ventral tegmental area connections to motor and sensory cortical fields in humans. Brain Struct Funct..

[44] Paxinos G., Watson C. (2007). The Rat Brain in Stereotaxic Coordinates.

[45] Galati S., Song W., Orban G., Luft A.R., Kaelin-Lang A. (2018). Cortical slow wave activity correlates with striatal synaptic strength in normal but not in Parkinsonian rats. Exp Neurol..

